# Natural Killer Cell Mechanosensing in Solid Tumors

**DOI:** 10.3390/bioengineering11040328

**Published:** 2024-03-28

**Authors:** Suzanne Lightsey, Blanka Sharma

**Affiliations:** J. Crayton Pruitt Family Department of Biomedical Engineering, University of Florida, Gainesville, FL 23610, USA; suzannelightsey@ufl.edu

**Keywords:** natural killer cells, mechanosensing, tumor microenvironment

## Abstract

Natural killer (NK) cells, which are an exciting alternative cell source for cancer immunotherapies, must sense and respond to their physical environment to traffic to and eliminate cancer cells. Herein, we review the mechanisms by which NK cells receive mechanical signals and explore recent key findings regarding the impact of the physical characteristics of solid tumors on NK cell functions. Data suggest that different mechanical stresses present in solid tumors facilitate NK cell functions, especially infiltration and degranulation. Moreover, we review recent engineering advances that can be used to systemically study the role of mechanical forces on NK cell activity. Understanding the mechanisms by which NK cells interpret their environment presents potential targets to enhance NK cell immunotherapies for the treatment of solid tumors.

## 1. Introduction

Despite significant advancements in understanding cancer development and progression, cancer remains the second leading cause of mortality [1]. In recent decades, cell immunotherapies have emerged as an asset in cancer treatment, especially for patients who have exhausted all other treatment options. Adoptive transfer of engineered chimeric antigen receptor (CAR) T cells has shown remarkable effects on inducing long-term remission in patients with hematological malignancies [2,3]. However, the application of these therapies is limited by severe side effects, variability in patient response, and challenges with cell sourcing [4,5,6]. As such, natural killer (NK) cells are an appealing alternative cell source for immunotherapies to circumvent these limitations. NK cells are innate lymphoid cells that play a crucial role in eliminating cancerous cells. Exciting advancements have been made in targeting hematological cancers using NK cell immunotherapies [7,8,9,10], but like most cell therapies, NK cell immunotherapies have proven less effective in treating solid tumors [11,12,13], which constitute ~90% of adult cancers [1]. For NK cells to exert their cytotoxic functions, direct contact with cancer cells is needed; however, NK cells must first traverse from blood or lymphatic vessels and migrate through a dense tumor matrix before interacting with their target cells. As such, NK cells that extravasate to the tumor largely remain in the stroma of solid tumors and only a small fraction come into direct contact with tumor cells [14]. In many cancer types, patients with low counts of tumor-infiltrating NK cells have a worse prognosis [15,16]. Therefore, identifying and overcoming the barriers within the solid tumor microenvironment that impede the efficacy of NK cell therapies is a necessary clinical imperative.

To address this imperative, the process through which cells are capable of sensing and responding to forces in their environment, i.e., mechanosensing, must be considered. Since the late 1800s, cell mechanosensing has been well-established in adherent cells [17]; however, the role of mechanosensing by NK cells has been largely overlooked and is only in the early stages of investigation. Understanding NK cell mechanosensing may lead to new insights regarding the molecular events in immune evasion during cancer progression and open avenues for engineering novel therapeutic targets for NK cell immunotherapies. This review provides an overview of current insights into NK cell mechanosensing, incorporating knowledge drawn from mechanical mechanisms in other relevant cell types to highlight opportunities for further exploration in NK cells. Furthermore, in the context of the solid tumor microenvironment, we examine the mechanobiological barriers present and discuss advanced engineering strategies for studying NK cell mechanosensing.

## 2. Natural Killer Cells

NK cells are the effector cells of the innate immune system and, as their name suggests, are capable of detecting and lysing cancer cells without prior antigen priming [18]. Unlike T cell activation, which relies on the knowledge of a specific antigen, NK cell activation uses a balance of inhibiting and activating signals from target cells. Although there is an amassing body of work defining novel NK cell phenotypes, this review will mainly focus on cytotoxic, CD56^dim^CD16^bright^ NK cells, which represent >90% of all NK cells, and NK92 cells, a clinically utilized NK cell line that lacks almost all inhibiting receptors [19,20]. Under healthy, physiological conditions, NK cells are inhibited by the expression of major histocompatibility complex I (MHC I); however, cancer cells down-regulate expression of MHC I and upregulate other cell surface ligands [21]. For NK cell activation to occur, strong stimulatory signals are required to overcome the inhibitory signals. Once activated, NK cells can destroy cancer cells directly through the release of membrane-destroying granules and modulate the cytokine milieu in the tumor microenvironment through interferon-γ (IFN-γ) production [22]. Because NK cells require strong activating signals from target cells, there is less risk that they will attack healthy cells in the body that almost ubiquitously express MHC I. Thus, both autologous and allogeneic adoptive NK cell therapies have excellent safety profiles [23].

## 3. NK Cell Mechanosensors

Mechanosensors allow cells to detect and respond to the physical forces within their surroundings. By activating mechanosensors on the surface of cells, the cell receives and transmits the signals inward, initiating a cascade known as mechanotransduction. This series of events dictates the cells’ capacity to interact with target cells. Mechanosensors are essential for many cellular processes, such as solid tumor progression [24], inflammation [25,26], and NK-target cell interactions [27,28]. Here, we will define the major components (i.e., integrins and ion channels) and locations (i.e., the immunological synapse and the nucleus) involved in NK cell mechanosensing and discuss their role in NK cell functions.

### 3.1. Integrins

Integrins mediate bidirectional signaling between the cells’ interior (cytoskeleton) and the cells’ exterior (e.g., other cells and extracellular matrix). These receptors not only play a crucial role in cell adhesion, but they also respond to mechanical signals of the tumor microenvironment by altering their protein conformation [29]. Integrins consist of two noncovalently associated subunits: an α-subunit and a β-subunit. In mammals, there are 18 αsubunits and 8 β-subunits, forming 24 different integrin heterodimers [30]. The mix of integrins results in a unique signature, with various binding affinities, that directs cell–matrix interactions such as adhesion and migration, as well as general cell functions such as survival and proliferation [31,32,33]. Forces applied to the cell surface pull open integrins, triggering their activation [34]. The receptors then transmit the mechanical forces from the cell membrane to the actin cytoskeleton by engaging with actin-binding adaptor proteins, otherwise known as clutch molecules, examples of which include talin and actinin [35,36]. Mechanical stress (i.e., the shear flow of blood, the adhesion force of other cells during immunological synapse formation, internal forces through clutch molecules on the cytoskeleton, etc.) can cause integrin–ligand bond strengthening [37,38]. Dysregulation of integrins is associated with tumor growth, invasion, and therapy resistance in many solid cancer types [39]. As such, integrins are promising targets for therapeutic intervention. However, integrin-targeted therapies focused on disrupting integrin interactions on cancer cells have been insufficient for the treatment of cancer patients thus far [40,41,42,43,44,45,46,47,48,49]. This can largely be attributed to the multifaceted role of integrins, where in some cancer types integrins have antitumor functions, and to the high levels of toxicity associated with integrin-targeted therapies. Therefore, novel approaches are needed to realize the potential of integrin-targeted therapies, such as targeting integrins on other cells in the tumor microenvironment, e.g., inflammatory/immune cells. While targeting leukocyte integrins has proven effective in treating autoimmune diseases, its application in cancer treatment is largely unexplored [50]. Indeed, adjusting the affinity of integrin binding is important for tuning the migratory capacity of cells, and, as such, strategies targeting integrins may be used in immunotherapies to modulate immune cell functions [25]. Therefore, understanding the biology of immune cell integrins and their relevant downstream molecules is necessary to modulate the immune response in solid tumors and discover therapeutic targets.

Human NK cells express lymphocyte function-associated antigen 1 (LFA-1, α_L_β_2_), macrophage-1 antigen (Mac-1, α_M_β_2_) in circulation, as well as very late antigen 4 (VLA-4, α_4_β_1_), VLA-5 (α_5_β_1_), and VLA-6 (α_6_β_1_) [51,52,53]. β_2_ integrins, particularly LFA-1, are more commonly associated with the adhesion of circulating NK cells to endothelial cells [32]. LFA-1 binds primarily to intercellular adhesion molecule 1 (ICAM-1) and contributes to the formation of the cytotoxic immunological synapse on target cells [32,54]. By facilitating the binding of NK cells to target cells and autonomously activating NK cells, LFA-1 is essential for effective killing. On the other hand, β_1_ integrins, exemplified by VLA-4, which binds primarily to vascular cell adhesion molecule 1 (VCAM-1), are often linked to tissue residency and interactions within the tissue microenvironment [32]. Both LFA-1/ICAM-1 and VLA-4/VCAM-1 interactions are critical for the extravasation and infiltration of recruited NK cells to the tumor [55,56]. Although it has not been directly explored in NK cells, in migrating T cells, it has been shown that VLA-4 contributes to cell rolling and LFA-1 is essential for crawling under shear flow [57]. Together, these integrins demonstrate a bistable mechanism that triggers upstream or downstream mechanotaxis in immune cells and may elucidate strategies to increase immune cell extravasation from the blood vessel [57]. Additionally, allosteric activation of VLA-4 and LFA-1 integrins in T cells using a small molecule, 7HP349, enhanced T cell infiltration into colorectal and melanoma tumors [58]. Despite the exciting advancements shown in T cells, the collaborative function of integrins, like LFA-1 and VLA-4, expressed on an NK cell remains an open question.

While integrins can bind to receptors on other cells, more commonly integrins bind to extracellular matrix (ECM) components. The ECM is an intricate network of proteins (e.g., collagens, laminins, fibronectin) and polysaccharides that provides structural support for cells. The ECM can vary vastly between tissues and, depending on the 3D structure that the ECM network forms, differing binding sites become available for integrins, thus altering cell behavior [59]. Notably, tissue-resident NK cells have differential expression of integrins compared to circulating NK cells, irrespective of the cell’s origin. This suggests that integrins and the binding sites available in the ECM may either direct precursor cells to, or retain mature NK cells in, these environments [60,61]. Although integrins play an important role in mediating NK cell adhesion, signal transduction, and immune synapse formation, it is important to note that some immune cells have evolved alternative mechanisms to respond to mechanical cues. For example, studies have reported that integrins may be dispensable for NK cell migration against viral infection [62]. Nonetheless, further research is needed to understand how different integrins influence NK cell infiltration and activation and thus the overall antitumor immune response.

### 3.2. Mechanically Gated Ion Channels

In addition to integrins, cells also rely on ion channels to respond to mechanical stresses. When cells are at rest, an electrochemical gradient across the cell membrane is maintained through a combination of passive diffusion and active ion transport. The regulation of transport by gated ion channels is contingent on specific chemical or physical stimuli, determined by the gating mechanism of the channel [63]. Activation of mechanically gated ion channels occurs through physical stimuli, which are believed to be perceived through ion channel-cytoskeletal interactions [63]. Piezo1 is one of the most widely studied mechanically gated ion channels. The activation of Piezo1 results in calcium cations entering the cells from the ECM. Calcium cations play a critical role in immune cell surveillance and signaling [64,65]. In T cells, Piezo1-mediated mechanosensing of fluid shear stress enhances calcium cation intake and therefore T cell activation through the calcineurin-NFAT transcription factor pathway [66]. In addition, optimal T cell receptor signal transduction requires Piezo1 activation at the immunological synapse, which may be facilitated by Piezo1-mediated calcium influx [67]. Furthermore, in innate immune cells, Piezo1-mediated mechanosensing assumes a pivotal role in triggering a pro-inflammatory response and modulated macrophage polarization [68,69].

In 2024, Yanamandra et al. pinpointed Piezo1 as the mechanosensitive ion channel primarily expressed in NK cells [70]. Activation of Piezo1 promotes calcium influx in NK cells, which is necessary for optimizing granule exocytosis and cytokine production [71,72,73]. As such, disrupting the function of Piezo1 resulted in the loss of sensitivity to stiffness-related cues thus significantly impairing NK cell-killing efficiency and infiltration. Interestingly, disrupting the function of Piezo1 did not alter the expression of cytotoxic proteins such as perforin and granzyme B, but rather notably reduced the number of NK cells that infiltrated into a 3D matrix. Conversely, activating Piezo1 improved NK cell killing and facilitated their migration within 3D collagen matrices [70]. These findings demonstrate the importance of mechanically gated ion channels for NK cell functions. Further research is needed on other mechanosensing ion channels in NK cells, such as transient receptor potential vanilloid (TRPV) channels, which are known to mediate T cell activation and cytokine production [63]. Investigating mechanically gated ion channels in NK cells in the context of tumor inflammation will enhance our understanding of NK cell inactivation and exhaustion and may reveal novel opportunities to rescue and/or prime autologous NK cells.

### 3.3. NK Immunological Synapse (NKIS) Formation and Cytoskeletal Rearrangement

**Figure 1:**
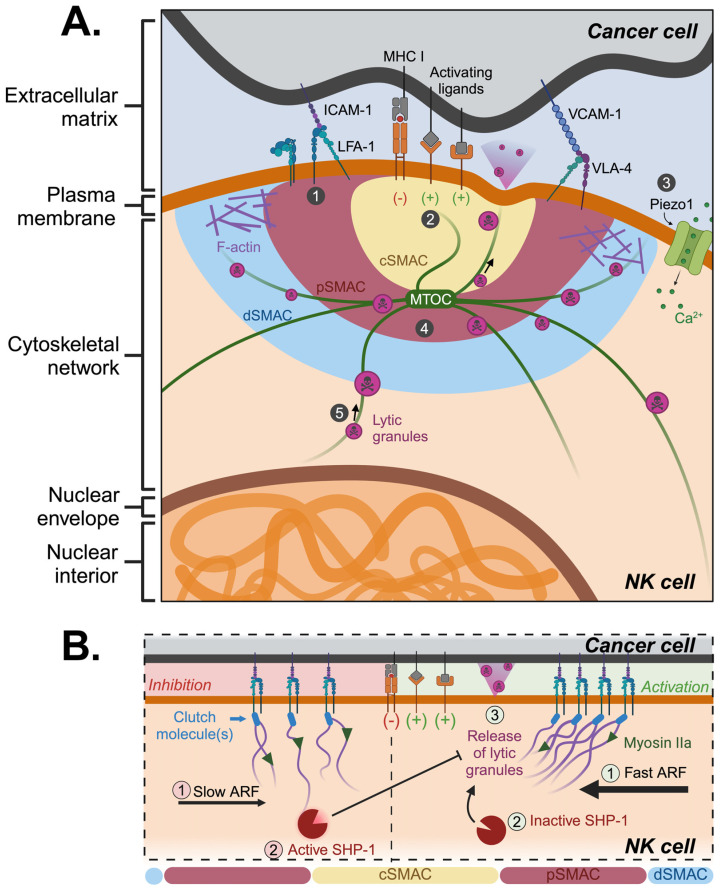
Mechanical mechanisms involved in NK immunological synapse formation. (**A**) NK cells use adhesion integrins to bind and interact with target cells. Upon binding, a signaling cascade results in cytoskeletal rearrangement and transport of lytic granules. The formation of the NKIS involves: (1) integrin adhesion, (2) recognition of activating ligands on the surface of cancer cells, (3) activation of Piezo1 leading to the influx of calcium ions, (4) cytoskeletal rearrangement with MTOC reorientation towards the cSMAC and F-actin accumulation towards the pSMAC, and (5) transportation of lytic granules to the target cell. (**B**) Following ligand engagement, fast actin retrograde flow results in NK cell activation whereas slow actin retrograde flow activates SHP-1 which inhibits the release of lytic granules. Created with BioRender.com.

Mechanosensing plays an important role in the release of cytotoxic granules to destroy target cells. When NK cells recognize a target cell, they form an NK immunological synapse (NKIS), which is the interface between the target cell and the NK cell (Figure 1A) [74]. The NKIS has a target-like configuration characterized by supramolecular activation clusters (SMAC) situated in central (cSMAC), peripheral (pSMAC), and distal (dSMAC) regions [74]. The inhibiting and activating receptors are concentrated within the cSMAC, while the pSMAC facilitates adhesion between the effector cell and the target cell via adhesion integrins like LFA-1 and MAC-1 [75]. Following the formation of NKIS, the resulting signaling cascade leads to cellular cytoskeleton remodeling with actin filament (F-actin) accumulation at the pSMAC and microtubule organizing center (MTOC) reorientation toward the cSMAC [27,75]. Although dysregulation of cytoskeletal contractility did not impact lytic granule conjugation, it resulted in increased actin density at the membrane, thereby significantly restricting the process of degranulation [74]. On the cell surface, activating cell surface receptors, such as NKG2D, NKp44, NKp30, and NKG2C, signal the polarization of MTOC. Mechanistic studies suggest that these receptors activate JNK (Jun N-terminal kinases) and ERK2 (extracellular signal-regulated kinase 2) which are responsible for MTOC polarization. To elaborate, the binding of activating receptors triggers Src family kinase activation of PI3K (phosphoinositide-3 kinase), leading to the activation of ERK2 and PLCγ. Subsequently, this sequence activates JNK downstream leading to cell polarization [76,77]. After polarization, microtubules form a highway to guide the movement of lytic granules towards the NKIS. Simultaneously, myosin IIa, a molecular motor protein, uses contraction forces along F-actin to traffic lytic granules. Upon reaching the NKIS, granules fuse with the plasma membrane, resulting in the release of perforin and granzymes. Through LFA-1, the synapse establishes a tight seal to promote granule-mediated cytotoxicity and restrict diffusion. Perforin monomers aggregate in the presence of calcium ions, forming pores in the target cell membrane. This enables granzymes to enter the cell, subsequently leading to the lysis of the adhered target cell [78]. Following the execution of their effector functions, NK cells endocytose the lytic granule membrane proteins to recycle the granules for further killing [27]. For further information on how the cellular cytoskeleton dynamics regulate NK cell functions, the authors direct the reader to a comprehensive review by Wong and Ding [28].

During NK cell polarization, the actomyosin dynamics are responsible for mechanotransduction in NK cells. Actin retrograde flow (ARF) is a cellular process characterized by the rearward movement of F-actin within the cell, driven by contractile myosin forces pulling actin filaments away from the cell periphery (Figure 1B). The velocity of ARF plays a crucial role in determining the balance between NK cell inhibition and activation [79]. Rapid lamellipodial ARF is associated with activating NKIS, while inhibitory NKIS exhibits slower ARF. Specifically, during slower ARF, tyrosine-protein phosphatase (SHP)-1 is activated and subsequently inhibits NK cell activation by dephosphorylating necessary signaling molecules, such as the guanine nucleotide exchange factor VAV1 [80] and the transmembrane adaptor protein LAT (linker for activation of T cells) [81]. During rapid ARF, SHP-1 remains in an inactive conformation and does not engage with the actin machinery, resulting in NK cell activation [82]. While the formation of NKIS is a relatively slow process, taking minutes to hours to complete, the dynamics of actin flow alterations and subsequent changes in SHP-1 status are rapid events, occurring within seconds. This swift modulation enables a quick and precise regulation of inhibitory signaling in NK cells [83]. Thus, controlling actin dynamics via ARF represents a novel mechanotransduction mechanism to regulate NK cytotoxicity.

### 3.4. Mechanically Induced Nuclear Deformation

For NK cells to reach tumor cells, they must extravasate from blood vessels and transmigrate into and within the tumor microenvironment. These spaces range from 1 to 20 μm in diameter, requiring the cell to squeeze through available spaces, which can alter intracellular organelles [84,85]. As the largest and most rigid organelle within eukaryotic cells, the nucleus is subjected to intrinsic (e.g., cytoskeletal rearrangement) and extrinsic forces (e.g., transmigration) that lead to nuclear deformation. Evidence indicates that the nucleus serves not only as the primary site for gene replication but also as an essential mechanotransduction component within the cell. The nucleus possesses the capability to sense mechanical stimuli and instigate dynamic changes in a cell’s structure and morphology [86,87]. A comprehensive review by Kalukula et al. describes the mechanisms of nuclear deformation and mechanoregulation and its associated roles in cellular mechanobiology [88].

The role of the nucleus as a mechanosensitive organelle has only begun to emerge and largely focuses on cells that have force transduction focal-adhesion complexes. As such, research on NK cell nuclear mechanoregulation and its impact on cell function is lacking. Nevertheless, observations made in other cell types offer insights that might be relevant to NK cells. For example, nuclear deformation induced by T-cell activation correlated with changes in gene expression, particularly in CD69 expression [89]. CD69, an early activation marker for T-cells, relies on ERK and NF-κB (nuclear factor-κB) signaling and is essential for NK cell-mediated killing of difficult-to-treat targets. In NK cells, transcription factor Eomes (Eomesodermin) and nuclear localization via intracellular contractility were associated with enhanced cytotoxicity against non-small cell lung cancer [90]. Therefore, NK cell nuclear deformation may induce changes to gene expression for immune activity and cytotoxicity, resulting in opportunities to regulate NK cell functions during transmigratory events. However, more work is required to determine the mechanisms behind nuclear deformation and its interactions with biological molecules in NK cells before its therapeutic potential can be realized.

Beyond influencing the cell’s gene expression, deformation of the cell’s nucleus may also impact its migratory potential. Seirin-Lee et al. demonstrated that nuclear deformation caused nuclear architecture reorganization and subsequently drove long-range migration in a mouse embryonic stem cell study [91]. Further, Krause et al. demonstrated that nucleus deformation in a 3D environment resulted in faster and more sustained migration in fibrosarcoma cells [92]. It is feasible that transmigration of NK cells through confined areas may be propelled by the forces generated during nuclear deformation. This is supported by a study demonstrating that dendritic cell migration through complex environments was facilitated by actin polymerization, driven by Arp2/3 (actin-related proteins 2/3), a process also involved in NK cell cytoskeletal rearrangement [93,94]. The studies discussed here indicate that nuclear deformation may preferentially influence NK cell activation and trafficking through the tumor microenvironment; however, research on the consequences NK cell nuclear deformation may have on NK cell migration, activation, and exhaustion warrants further investigation.

## 4. Physical Traits of Solid Tumors

**Figure 2:**
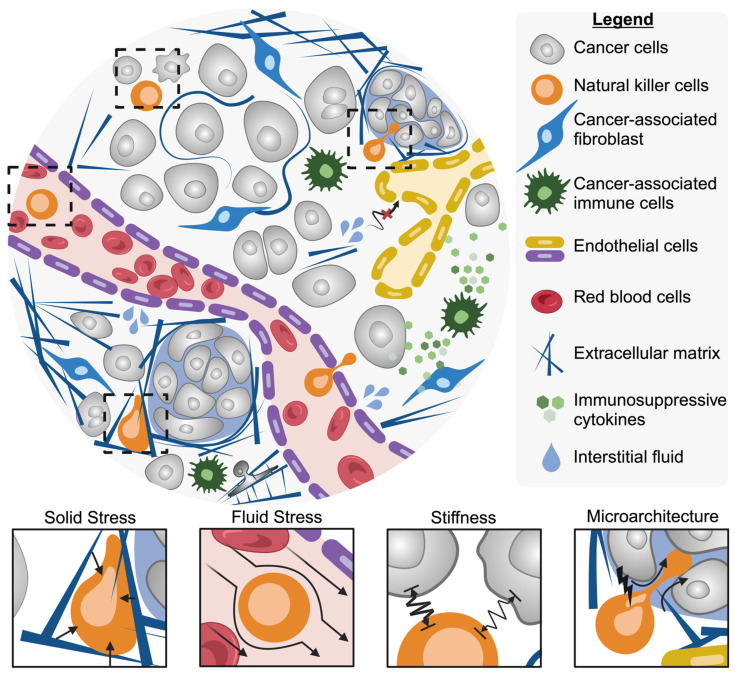
The physical traits and stresses that NK cells encounter in solid tumors, including solid stress, fluid stress, altered cell stiffness, and altered microarchitecture. Created with BioRender.com.

Tumor cells manifest distinct physical, chemical, and genetic features compared to healthy tissue. These features can be used to define the tumor’s classification, develop treatment strategies, and determine patient prognosis. Tumors are commonly classified as either solid (i.e., carcinoma, melanoma, sarcoma, etc.) or nonsolid (i.e., hematological neoplasms or blood cancers). This classification is based on the presence or absence of a liquid area within the tumor structure. Historically, research on anti-solid tumor therapies largely focused on the biological abnormalities of solid tumors [95]. Exploration of the physical forces involved in cancer progression has led to significant findings, uncovering previously unexplored strategies for treatment. The physical traits of cancer are described by Nia et al. as solid stress, fluid pressure, stiffness, and microarchitecture (Figure 2) [96]. Understanding the influence and consequences the physical traits of solid tumors have on NK cell activity is necessary for improving treatment.

### 4.1. Elevated Solid Stress

In solid malignancies, tumor cells are both subject to and producers of mechanical forces resulting in stresses. Solid stress can be further classified into two parts: inside the tumor [97] and at the tissue interface [98]. Stress inside the tumor, otherwise known as residual stress, is the microscopic force among the structural components of the tumor microenvironment. Residual stresses remain in the tumor even after the tumor has been separated from the surrounding tissue [99]. This stress arises as cells proliferate and migrate, exerting forces on solid components of the surrounding matrix. In addition, the expansion of existing ECM components, such as hyaluronic acid, through osmotic water adsorption causes further stress [100,101]. Stress at the tissue interface is described as externally applied stress from the surrounding, healthy tissue to inhibit tumor expansion. The applied external force can be large enough to compress blood and lymphatic vessels in and around tumors which impairs blood flow and thus the delivery of oxygen, therapeutic drugs, and immune cells [99]. In vitro, tumor spheroids cultured under osmotic pressure have decreasing compressive stress from the center to the periphery, and mechanically confining the spheroid hindered its growth [102]. Despite the development of various methods, accurately measuring the magnitude and distribution of solid stress remains a technical challenge in vivo.

Solid stress equips cancer cells to evade the immune system by activating the PI3K/Akt pathway. Tumor cell surface integrins, primarily VLA-3, interpret the physical forces and cause changes in the downstream genes STAT3 (signal transducer and activator of transcription 3) and MYBL2 (myb proto-oncogene like 2), leading to overactivation of the PI3K/Akt pathway [103]. The upregulation of Akt in cancer cells can result in the upregulation of inhibitory, anti-apoptotic molecules, thus making the cells more resistant to NK cell-mediated killing. Solid stress levels differ in primary and metastatic tumors indicating a relationship between solid stress and invasion of cancer cells [102]. Although the reason for this difference is still under investigation, this relationship may be partially explained through the Hippo signaling pathway. The Hippo signaling pathway consists of YAP (Yes-associated protein) and TAZ (transcriptional coactivator with PDZ-binding motif) which are potent mechanoresponsive factors that, in response to physical cues such as compression by cell crowding, translocate from the cytoplasm to the nucleus [104,105]. YAP/TAZ promotes a therapeutic-resistant and metastatic tumor cell phenotype. Additionally, YAP/TAZ is associated with epithelial–mesenchymal transition (EMT) of cancer cells which promotes the invasion of cancer cells [106].

Currently, there is no research on the impact of solid stress on NK cell mechanotransduction pathways. However, it has been demonstrated that intracellular contractility inactivates TAZ to promote NK cell cytotoxicity towards cancer cells, providing a potential strategy for the ex vivo rejuvenation of autologous NK cells [107]. In addition, the physical consequence of solid stress poses a significant barrier to NK cell infiltration and survival in solid tumors. As previously mentioned, externally applied stress can compress blood vessels resulting in a limited supply of nutrients and oxygen. Vessel compression also contributes to hypoxia and the accumulation of reactive oxygen species [91]. NK cells are particularly sensitive to inactivation and cell death from the build-up of reactive oxygen species, more so than T and B cells [108]. As such, a deeper understanding of the mechanisms and influence of solid stress on natural killer cell antitumor functions is needed.

### 4.2. Elevated Fluid Stress

Fluid stresses are the forces exerted by the fluid elements within the tumor. These include the interstitial fluid pressures (IFP) and shear stress exerted by blood and lymphatic vessels. Of note, solid stress can induce elevated fluid stress, and vice versa, but these mechanical stresses are independent with differing origins and downstream results [109]. 

#### 4.2.1. Interstitial Fluid Pressure (IFP)

At homeostasis, arteries transport blood to tissues, and veins carry it away, while lymphatic vessels drain excess tissue fluid resulting in near-zero IFP. However, as discussed in the section above, solid stresses compress vessels within the tumor causing leakage from abnormally permeable blood vessels and inadequate lymphatic drainage [96]. This results in an accumulation of interstitial fluid which further reduces tumor perfusion. Ultimately, solid tumors exhibit elevated IFP, ranging from <1 kPa in brain tumors to 5 kPa in renal cell carcinomas [96]. Within a tumor, IFP is fairly uniform and drops abruptly at the tumor periphery, generating a steep pressure gradient that causes fluid to leak into the surrounding tissue [110]. Although IFP causes cancer and tumor-associated cells to create an immunosuppressive cytokine milieu, IFP is primarily a major barrier to drug transport. Given that IFP can reach levels comparable to microvascular pressure, the main mechanism of drug delivery is through diffusion. An increased IFP leads to decreased capillary transport due to vessel compression and, because of the steep pressure gradient, fluid flows from the interior of the tumor to the periphery [111]. Therefore, minimal drugs are delivered and those that are delivered are pushed out of the tumor, resulting in a reduced therapeutic efficacy.

As IFP is caused by fluid abnormalities in solid tumors, therapeutic strategies have focused on vasculature normalization [112]. Vascular normalization involves the restoration of leaky and tortuous vessels to a more functional state thereby reducing IFP. In the clinic, drugs such as bevacizumab, an antibody targeting vascular endothelial growth factor A (VEGF-A), along with inhibitors for VEGF receptor tyrosine kinases, are approved for the treatment of over a dozen types of solid tumors [113]. Beyond lowering IFP in solid tumors, anti-VEGF treatments increase the amount of effector cell infiltration and sustain immune cell survival [112].

#### 4.2.2. Vascular Shear Stress

Fluid flow within vessels produces a shear force on the inner surface of the blood vessel. Tumor blood vessels have irregular structures and varying vessel diameters due to IFP and excessive VEGF production, creating shear stresses that vary widely within the vessel [114,115]. NKG2D-mediated mechanosensing of fluid shear stress enhanced NK cell activation and degranulation [116]. Although not directly linked to fluid shear stress, tumor-associated endothelial cells down-regulate genes related to immune extravasation, such as ICAM-1 and VCAM-1, in many cancers, further contributing to a less permissive environment for NK cell extravasation to the tumor tissue [117,118]. Interestingly, under low fluid shear, neutrophils can directly bind to tumor cells. Bound neutrophils can both traffic the tumor cells across the endothelium through ICAM-1-β_2_ integrin interactions and act as a shield for cancer cells [111,119]. This shielding mechanism serves to protect tumor cells from mechanical damage caused by shear stress and assists them in avoiding direct exposure to NK cell-mediated surveillance.

### 4.3. Increased Stiffness and Altered Mechanical Properties

Increased tissue stiffness of solid tumors is the best-recognized mechanical abnormality. Stiffness is a diagnostic marker for solid tumors [120], and more recently a prognostic marker [121,122]. Tumor stiffness is defined as the ability of a material to resist deformation when subjected to a force applied at a very slow rate. Unlike solid and fluid mechanical stresses, stiffness is an intrinsic material property of tumors. Tumor stiffening is primarily caused by the deposition and crosslinking of ECM components, such as hyaluronic acid and collagen, which are mainly secreted by cancer-associated fibroblasts. Ranging from 1 kPa in brain tumors to 70 kPa in cholangiocarcinomas [97], solid tumors are usually 5–10 times stiffer than healthy tissue [96,123]. Many studies have shown that the stiffness of the tumor microenvironment causes many of the traits central to cancer including proliferation, angiogenesis, altered metabolism, and increased cancer invasion [124,125,126,127,128,129,130,131,132,133]. Indeed, the stiffness of the tumor ECM changes at various stages of tumor development. For instance, in the development of lung adenocarcinoma, stage III tumors exhibit greater stiffness compared to stage II tumors [134]. While heightened tumor stiffness is associated with increased malignancy, there could be opportunities to leverage this physical property of tumor development using mechanosensitive treatments. The authors direct the readers to more detailed reviews on the influence of tumor material [135,136].

Interestingly, recent studies have revealed that stiffer substrates can increase NK cell-related killing. NK cells cultured on a rigid 2D substrate (142 kPa) demonstrated increased secretion of IFN-γ granzyme A and B, Fas ligand, and granulysin compared to those on softer substrates (1 kPa) [137]. Moreover, experiments by Friedman et al. involving surrogate cell-sized spherical beads with varying stiffness (9 kPa, 34 kPa, and 254 kPa) found that stiffer beads facilitated NK cell spreading, faster ARF, and degranulation [137]. Conversely, softer substrates hindered the polarization of the MTOC to the pSMAC, resulting in the formation of unstable synapses that induce slow ARF. This suggests that not only do stiffer substrates boost NK cell activation, but softer substrates also impede NK cell functions, thus presenting a ‘blind spot’ in NK cell killing. Of note, the engagement of LFA-1 and subsequent talin recruitment were not inhibited in soft matrices [137]. As such, combining a soft substrate with an activating signal (e.g., CD28) allowed NK cells to overcome slow ARF and partially restore their effector functions. In another study, it was demonstrated that the addition of PMA/ionomycin, an NK cell stimulant, reinstated NK cell cytotoxicity [82]. The ability to rescue NK cell activity on soft substrates suggests the existence of a downstream signaling cascade that has yet to be discovered in NK cells and emphasizes the importance of studying NK cell activity in a physiologically relevant system.

#### 4.3.1. Metastatic Cancer Cell Softening

Cancer cells with an increased potential for metastasis are relatively softer than normal cells, measured via atomic force microscopy (AFM) [138,139]. The influence of stiffness on the functions of NK cells, particularly in cell-eliminating processes, has only recently garnered attention. This attention was sparked by findings demonstrating that the effectiveness of NK cell-mediated elimination of target cells is influenced by the target cells’ stiffness [137]. Therefore, identifying methods to prevent or reverse tumor cell softening may aid immunotherapies. EMT has been suggested as a mechanism responsible for primary tumor softening and the facilitation of metastasis [140]. Manipulation of EMT transcription factors, such as TWIST and SNAIL, may delay the softening of cancer cells and give NK cells more time to form a stable NKIS and effectively eliminate the cancer cells. Of note, the tumor microenvironment is a complex ecosystem, and delaying cancer cell softening may also increase solid and fluid stresses present in the tumor, resulting in a worse prognosis. Alternatively, identifying methods to sensitize NK cells to softer target substrates may aid NK cells in overcoming inhibitory signals transmitted from target cells.

#### 4.3.2. Altered NK Cell Stiffness

It is established that the stiffness of NK cells directly influences their ability to navigate through tissues [141,142]. Despite having a diameter larger than some blood vessels, NK cells possess low shape compliance, making their extravasation a complex and challenging process. Medler et al. demonstrated that reducing NK cell rigidity through modulating the cytoskeletal organization enhanced tissue infiltration [141]. However, altering the rigidity of NK cells may jeopardize their effector functions, posing a double-edged sword. Indeed, intracellular contractility, which is associated with increased rigidity, is necessary to induce nuclear localization of various NK effector genes (e.g., NF-κB [143] and Eomes [90,144]). Therefore, striking a balance between NK cell rigidity and flexibility is needed.

Interestingly, IL2, a commonly used growth supplement for sustaining and expanding NK cells and cell lines in culture, was found to increase NK cell stiffness, an effect that persisted for up to 96 h following IL2 withdrawal [145]. Therefore, common assessments of NK cell functions in vitro should be interpreted cautiously, as they might not entirely reflect the in vivo dynamics due to the altered biophysical attributes of NK cells. Unbiased analysis will allow for the identification of crucial pathways that modulate NK cell rigidity which could be manipulated ex vivo before adoptive transfer into recipients.

### 4.4. Altered Tissue Microarchitecture

Healthy tissues are highly structured and heterogeneous. However, as tumors grow, both the tumor itself and the surrounding tissues are structurally disrupted, disturbing homeostasis [111]. Instead of being completely disordered, cancer-associated fibroblasts secrete ECM fibers, mainly collagen, that become dense and orderly, resulting in a packed matrix with confined pores [146,147]. The architecture of the local environment affects migration. In early breast tumors, collagen fibers are commonly arranged in a ring-like structure that encapsulates the tumor. However, in terminal breast tumors, the outer fibers are shifted so that they no longer surround the tumor but enter the tumor tissue tangentially creating a ‘highway’ along the direction of tumor cell infiltration [148,149]. Collagen alignment has been shown to modulate MMP-dependent mechanisms [150] and integrin β_1_ expression [151], thereby impacting cells’ ability to migrate. When cells are confined within the ECM causing membrane deformation, intercellular calcium ions enter the cytoplasm through the stretch-activated ion channel, Piezo1. This sequence of events leads to the suppression of protein kinase A activity, a regulator of tumor cell migration through GTPases, particularly RhoA and Rac1 [152].

Further, the dense crosslinking of ECM fibers results in a physical barrier that results in NK cells, and other large therapeutics (e.g., pembrolizumab), accumulating in the stroma surrounding the tumor without efficient contact with cancer cells [153]. For NK cells to infiltrate into the tumor, they must navigate through the narrow and compressed channels within the ECM. NK cells can exhibit a distinctive ‘amoeba-like’ migration pattern, enabling them to forcefully deform their nuclei to pass through these confined channels [154]. However, as mentioned previously, the nucleus possesses only a limited capacity to deform itself, and the pores in the ECM force the cell to adhere to the route of least resistance. Tumors can utilize this mechanism to further evade direct contact with NK cells. Repeated nucleus deformation results in DNA double-stranded breaks, chromosomal aberration, and genomic instabilities in both cancer and NK cells [155,156]. While this may promote cancer progression, the mechanical stress from deformation can result in damage to NK cell motility and even cell death [155]. As such, therapeutic strategies targeting the tumor microarchitecture or NK cell migration patterns may increase NK cell infiltration in solid tumors.

## 5. Advanced Engineering Approaches

The investigation of mechanical forces in immune cell functions is an area of intense research. Although the idea that mechanical forces influence cell behavior is not new, detecting mechanical forces within and on cells is challenging as these forces often operate on a nanometric length scale. This problem is exasperated in NK cells, which are nonadherent. Techniques such as optical tweezers, micropipettes, and AFM have been employed to detect such forces, but are often limited to a specific point on the cell membrane [157]. Traction force microscopy, which maps forces across entire cells by measuring the displacement of fluorescent beads in a hydrogel, provides a broader view; however, there are limitations in tracking the relocation of individual beads [158]. While these methods contributed to advancing the understanding of mechanosensing in leukocytes, the acquired information is predominantly limited to T cells and B cells. Le Saux and Schvartzman provide an in-depth review of advanced materials and technologies for the study and regulation of NK cells [159]. Here, we will further focus on devices for exploring NK cell mechanobiology. 

### 5.1. Microfabrication Techniques

Microfabrication enables control over the physical and mechanical cellular microenvironment. This allows the fabrication of microenvironments with defined stiffness, topography, and geometries to investigate how these factors influence NK cell adhesion, migration, and activation. Here, we will examine two microfabrication techniques: microfluidics, which can mimic fluid stresses, and micropatterning, which can mimic solid tumor microarchitecture.

#### 5.1.1. Microfluidics

Microfluidic devices can create controlled microenvironments to mimic physiological conditions. These devices enable the precise manipulation of fluid flow, shear stress, and the presentation of ligands to study NK cell responses. Recently, a tumor-on-a-chip device included breast cancer cells embedded in a 3D matrix, featuring a lumen at a distal end to simulate the vasculature and the shear forces found within the tumor. NK92 cells, a clinically relevant NK cell line, were then perfused through the lumen. From this study, Ayuso et al. initially observed rapid eradication of cancer cells by NK92 cells, but also found that extended exposure to cancer cells led to NK cell exhaustion [160]. Further, when breast cancer spheroids were embedded in a 3D collagen hydrogel within the microfluidic device, NK cell-induced cytotoxicity and antibody-dependent cellular cytotoxicity were induced at the spheroid surface [161]. Interestingly, NK cells penetrated the tumor spheroid faster than the antibody, suggesting that NK cells can squeeze through cancer cell–cell junctions and transmigrate through the spheroid. However, neither of the aforementioned studies investigated how the microenvironmental factors influenced NK cell activity.

NK cells interact with dendritic cells which are critical antigen-presenting cells that regulate adaptive immunity in vivo. The bidirectional crosstalk between NK cells and dendritic cells is necessary for coordinating antitumor responses [162]. However, the physical parameters associated with NK cell–dendritic cell interactions remain elusive. In a study conducted by Hipolito et al., two microfluidic devices were engineered to facilitate high-throughput analysis of NK cell migration properties and NK–dendritic cell interactions in vitro [163]. In this system, the authors demonstrated that mature dendritic cells interact with NK cells more often than immature dendritic cells, but immature dendritic cells more frequently come into contact with multiple NK cells [163]. The variations in NK–dendritic cell interactions and the strength of the subsequently formed NKIS may contribute to the diverse maturation of immature dendritic cells, offering insights for research strategies aimed at manipulating these interactions.

#### 5.1.2. Micropatterning

Creating spatially defined adhesive patterns on surfaces can help in studying how NK cells respond to specific mechanical cues. This technique allows for the control of cell–substrate interactions. Using micro-contact printing, Culley et al. alternated microstrips of NKG2D antibodies and a combination of NKG2D and inhibitory NKG2A antibodies and demonstrated that cell spreading and the formation of stable synapses in NK cells plated on these patterns were confined to the regions of NKG2D antibody only [164]. This study shed light on the cross-talk of LFA-1, the activating receptor NKG2D, and the inhibiting receptor NKG2A and how the balance of activating and inhibiting ligands is assessed by NK cells. Moreover, recent findings indicate that surface nanotopography and cell shape modulate cancer cell susceptibility to NK cell cytotoxicity [165]. NK cells demonstrated increased cytotoxicity against cancer cells on nanogrooved surfaces compared to cancer cells on flat surfaces. Additionally, the formation of stress fibers in cancer cells was found to correlate with NK cell-mediated killing, suggesting that the increased cellular tension in tumor cells, mediated by the nanogrooves, is a factor that regulates NK cell cytotoxicity [165]. Furthermore, immune cell shape can be controlled using a micropatterning approach. While not applied to NK cells, evidence indicates that cell elongation alone, without the addition of exogenous cytokines, led to an anti-inflammatory M2 macrophage phenotype [166]. This suggests that cell shape associated with the altered solid tumor architecture could influence the inflammatory response. Photolithography has also been used to generate surfaces with particular spatial arrangements of anti-CD3 antibodies and ICAM-1 for T-cell activation directly inspired by the “bull’s eye” morphology of the SMACs signaling [167]. Similar biologically inspired patterns can be applied to advance our understanding of methods for directing NK cells.

### 5.2. 3D Tissue Engineering

Tissue engineering involves the creation of three-dimensional (3D) structures that are capable of replicating the complexity of native tissues. Engineering culture systems and materials with tunable mechanical properties allow for the investigation of NK cell mechanosensing in a physiologically relevant system and can be leveraged to alter or restore NK cell functions.

#### 5.2.1. Hydrogels

Engineered 3D hydrogels can mimic the physiological environment more closely than 2D, providing a platform to study NK cell mechanosensing in a tissue context. Hydrogels can be made using native proteins (e.g., collagen or hyaluronic acid), ECM extract (e.g., decellularized extracellular matrix or Matrigel), or more advanced synthetic polymers (poly(ethylene) glycol (PEG) or polyacrylamide) [111,168,169]. Generally, synthetic polymers are more easily engineered to control the swelling, degradation, and porosity than natural materials. Within a hydrogel, the stiffness levels can be manipulated by adjusting the hydrogel composition, molecular weight, or concentration [111,168,170]. Of note, modulating stiffness through these factors may alter other properties of the material, such as porosity and degradation. Hence, achieving controllable mechanical properties may require simultaneous adjustments to multiple factors. Additionally, spatial confinement and the associated stresses can be studied by encapsulating cells in hydrogels with engineerable porosities.

We have developed a PEG-based hydrogel system in which biochemical adhesion and degradable sites can be incorporated without significantly varying the physical components [171]. Using this hydrogel system, we encapsulated either non-small lung cancer metastatic (H1299) or nonmetastatic (A549) cells and investigated their immunosuppressive milieu. The metastatic tumor model exhibited a more pronounced loss of stress ligands and a heightened production of inhibitory soluble molecules compared to the nonmetastatic tumor model, underscoring the physiological relevance of this 3D cancer model. Our group was the first to apply a synthetic, hydrogel system to investigate NK cell infiltration in 3D. Using this system, we found that the migration of NK92 cells towards cancer cells and their colocalization was contingent on the immunosuppressive milieu of the tumor models [171]. Beyond the release of biochemical cues, this model offers a platform to explore the impact of matrix stiffness on NK cell migration. In addition, Yanamandra et al. used bifunctionalized, poly (acrylamide-co-acrylic acid) hydrogels of varying stiffness to mimic target cells and demonstrated the stiffness-dependent NK cell responsiveness, with NK cells being more responsive on stiffer matrices [70]. Collectively, these studies demonstrate the use of hydrogels to further the understanding of NK cell mechanosensing.

#### 5.2.2. Microspheres

It was recently demonstrated that NK cells encapsulated in porous microspheres were protected from the physical and biological immunosuppressive tumor microenvironment effects. This has clinical significance as a major barrier to NK cell-based immunotherapy is ineffective NK cell infiltration and exhaustion due to the stress present in the solid tumor microenvironment. The microspheres were made of an alginate-poly(ethylene) oxide solution and had diameters ranging from 250 to 700 μm. Given the microporous structure, nutrient and oxygen exchange was feasible, enabling proliferation and cytokine secretion from encapsulated NK cells. Wu et al. illustrated that NK cells within the microspheres exhibited robust tumor cell killing in vivo through sustained release of perforin and granzymes and were able to initiate contact-dependent killing of surrounding target cells by budding from the microspheres [172]. It would be interesting to investigate if the mechanical deformation induced by NK cell budding contributed to the antitumor response. Although their antitumor effects have yet to be explored in detail, NK cells encapsulated in microspheres present a new strategy to sustain NK cell activity, mechanically activate NK cells, and potentially shield them from the physical stresses present in solid tumors.

### 5.3. Nanoscale Materials

Nanoscale materials serve as tools to probe and manipulate cellular processes at the molecular levels. These include nanowires, nanoparticles, and other nanomaterials, which offer exciting avenues to deliver biomolecules or modulate mechanical signaling pathways in NK cells.

#### 5.3.1. Nanowires

Nanowires, which are dense arrays of vertical rods, are gaining attention to study NK cell mechanobiology. Le Saux et al. synthesized nanowires via chemical vapor deposition, and then seeded NK cells on top of the arrays [173]. This platform employs nanowires with mechanical compliance and site-selective tip functionalization with antigens to generate both physical and chemical stimuli. Using this system, it was demonstrated that NK cells can exert approximately 10 pN of a pinching force in dense nanowires, leading to the immobilization of MICA, an essential step for NK cell degranulation [173]. Subsequently, the same research group showed that nanowires measuring approximately 20 μm in length could induce NK cell activation, even without antigen functionalization on the nanowires [174]. Together, these data underscore that nanowires are more effective at stimulating NK cells than 2D, flat surfaces.

Beyond activating NK cells via mechanical cues, nanowires can deliver biomolecules into the cytosol of NK cells. The nanowires’ high mechanical compliance allows precise tuning of their diameters and lengths. Moreover, their mechanical compliance facilitates the spontaneous penetration into the cell membranes. Biomolecules such as small interfering RNA (siRNA) can be deposited on the nanowires via plasma treatment. These coated nanowires have proven effective in delivering siRNA into cells without compromising their viability [175]. It is important to note that while this method can be used to deliver biomolecules to diverse cell types, the nanowires’ length and density would need to be carefully optimized for specific cells [175]. Primary NK cells are notoriously difficult to maintain in vitro after lentiviral/retroviral transduction. As such, the direct delivery of biomolecules into NK cells, bypassing the need for transduction, has immense translational potential.

#### 5.3.2. Nanoparticles

Nanoparticles are commonly leveraged for targeted delivery, imaging, and activation of NK cells. When compared with conventional drugs, nanomaterials can improve targeting and more accurately control the release of drugs, thereby reducing adverse, off-target reactions. Jiao et al. utilized gold nanoparticles conjugated with CD2 to label NK cells. These particles served both as a contrast agent for computerized tomography (CT) in vitro and a stimulant to enhance NK cell activity against melanoma and neuroblastoma cells [176]. Another research group proposed labeling NK cells with iron oxide nanoparticles functionalized with dopamine. Due to the magnetic properties of these iron oxide nanoparticles, an external magnetic field enabled the mechanical guidance of NK cells toward the tumor site. The labeled NK cells, propelled by the magnetic field, exhibited increased infiltration into solid tumors, and their in vitro and in vivo antitumor activity was bolstered by dopamine labeling [177]. Similarly, Sim et al. fabricated nanocomplexes from clinically available materials (hyaluronic acid, protamine, and ferumoxytol) for magneto-activation to enhance the activation of NK cells with the application of an exogenous magnetic field. Upon application of a magnetic field, NK cell-specific mechanosensing components such as NKG2D receptors and actin filaments were stimulated, and hepatocellular tumor growth was suppressed [178]. In addition, these nanocomplexes acted as a contrast agent for magnetic resonance imaging (MRI), enabling NK cell tracking in vivo. Furthermore, Lim et al. suggested using quantum dot particles to track NK cells delivered for cancer therapy [179]. A quantum dot is a nanoscale semiconductor particle that exhibits size-dependent optical properties, emitting light when excited. As such, researchers tagged human NK cells with quantum dots that were functionalized with the CD56 antibody. Although the quantum dots did not affect the in vivo antitumor therapeutic efficiency of NK cells, the quantum dots enabled effective near-infrared imaging of the tumor tissue [179]. Despite the translational potential of nanoparticles as delivery vehicles and contrast agents, the characteristics necessary for facilitating their uptake by NK cells are not well understood. This is especially important when delivering siRNA to NK cells, as a high dose of nanoparticle is needed to induce gene knockdown [180]. As such, it is necessary to explore further the nanoparticle features needed for effective uptake by NK cells to design more efficient delivery platforms for this cell population [180].

While exciting advancements have been made in the development of novel materials for NK cell trafficking and activation in vivo, critical NK cell–nanomaterial relationships need to be explored further. For example, the physical forces that nanoparticles exert on NK cells’ cytoskeletal network and its functional implications remain unknown. The cytoskeleton is essential for nanoparticle uptake and intracellular transport [181]. However, some studies report that lung cells exposed to nanoparticles had disrupted cytoskeletal functions which caused decreased proliferation, migration, and in some cases, cell death [182,183]. While this may be an effective strategy for maintaining tumor growth, the dysregulation of NK cells’ cytoskeletal function could render the cells functionally inert. These studies underscore the importance of thoroughly investigating the interactions of nanomaterials within immune cells, which remains largely unexplored, to ensure their safe applications in medicine. Nevertheless, the potential of leveraging nanoparticles to manipulate mechanosensing within a cell remains an exciting prospect for exploration.

#### 5.3.3. Backpacks

Polymer micropatches, also known as backpacks, present a materials-based strategy for locally activating NK cells, aiming to enhance the effectiveness of adoptive NK cell therapy. Prakash et al. introduced micropatches as cell engagers (MACE) designed to stimulate the natural cytotoxicity receptors, NKp30 and NKp46, on NK cells. This activation occurs while preserving the cells’ viability, migration capacity, and tumor-killing potential [184]. The discoidal geometry of MACE allows for localized microscale clustering of receptors, influencing cell activation status. It is conceivable that the mechanical force of MACE on NK cell receptors contributes to the greater signaling observed compared to free antibodies. This technology needs a minimal amount of antibody for NK cell activation, potentially reducing the cost associated with administrating large quantities of antibodies. While questions regarding the safety profile of this technology persist, these polymer patches exhibit promising therapeutic potential due to their scalability, deployability, and potential synergy with existing priming methods in the clinic. 

## 6. Conclusions and Outlook

Immunotherapy, particularly NK cell therapy, holds considerable promise as a cancer treatment, evident from the numerous candidates undergoing clinical trials. Addressing the physical challenges posed by the tumor microenvironment is pivotal for enhancing NK cell delivery and optimizing their antitumor functions. A comprehensive understanding of how NK cells perceive their surroundings and the mechanisms through which tumors exploit mechanosensing pathways to evade detection is imperative for enhancing therapeutic efficacy. Despite the advancements detailed in this review, the study of NK cell mechanobiology is still in its infancy. The absence of physiologically relevant models for assessing the impact of mechanical forces on NK cells within solid tumors underscores the need for cautious interpretation of findings derived from conventional in vitro testing. To address this gap, we have outlined advanced engineering approaches that may provide more accurate insights into mechanobiology. Future investigations into the interplay between solid tumor mechanics and NK cell immune response are poised to yield invaluable insights, fostering enhanced NK cell infiltration and bolstering their antitumor functions.
